# Comparisons of factors associated with suicidal ideation of older individuals by health status

**DOI:** 10.1093/geroni/igab046.3374

**Published:** 2021-12-17

**Authors:** Hyeyeon Sung, Jihun Na, Sungkyu Lee, Sehyun Baek

**Affiliations:** 1 Soongsil University, Seoul, Seoul-t'ukpyolsi, Republic of Korea; 2 Korea Center on Gambling Problems, Seoul, Seoul-t'ukpyolsi, Republic of Korea

## Abstract

This study examined the factors associated with suicidal ideation among older individuals and compared those factors by their objective and subjective health status. Data were obtained from the 13th wave of the Korean Health Panel Survey in 2018. The sample of 6,283 older individuals, who are 55 years and older, was classified into four groups by their objective and subjective health status. Objective health status was measured by the number of chronic health conditions, and subjective health status was defined by an individual’s self-reported health status. To examine the factors associated with suicidal ideation among four groups of older individuals, logistic regression analyses were conducted after controlling for socio-demographic characteristics, physical health and mental health characteristics. The results show that depression and anxiety were found as common factors associated with suicidal ideation for all four groups. As for the group of older individuals who reported bad objective health and bad subjective health, younger age, being male, and low educational attainment were found to be associated suicidal ideation. For those with bad objective health regardless of their subjective health status, the level of stress was found to be related to suicidal ideation among older individuals. Based upon those results, the present study discussed practical and policy implications for suicide prevention among older individuals by reflecting their objective health and subjective health status.

